# Medical legal validity of the use of the anomaloscope in the dyschromatopsia of aspiring civil and military aircraft pilots

**Published:** 2020

**Authors:** Mauro Salducci, Arianna Deandri

**Affiliations:** * Department of Sense Organs, Faculty of Medicine and Dentistry, Medical Clinic in Forensic Ophthalmology, Sapienza University – Umberto I Hospital, Rome, Italy

**Keywords:** dyschromatopsia, anomaloscope, CAD

## Abstract

Color blindness is a condition of altered color perception, scientifically defined as “dyschromatopsia”. Color blindness affects 8% of the world population.

Color blindness is caused by an alteration of the cones that influences the vision of the color self (red, green, blue). A comparative study was conducted in dischromatopsic subjects identified during the course of the ordinary investigations directed towards the civil aero-navigating personnel by the Ophthalmology Department of the Air Force, between March 2019 and January 2020, at “Aldo Di Loreto” Institute of Aeronautical and Space Medicine of Rome. 10 subjects aged 20 to 50, with dyschromatopsia found at Ishihara’s pseudoisochromatic tables, were submitted to Oculus HMC-Anomaloscope with a manual execution program and then a CAD test. Thus, in 2 out of 10 cases of dyschromatopsia, the Anomaloscope would have guided the medical judgement, while the CAD test would have oriented towards a judgment of full fitness despite the same lack of chromatic sensitivity however, underlined by both tests. In conclusion, the CAD test confirmed a highly sensitive and specific method of determining the presence and depth of the chromatic perception deficit but also the method was able to prevent the unjust refusal of certain air navigation activities to the aspirant staff.

## Introduction

Color blindness is a condition of altered color perception, scientifically defined as “dyschromatopsia”. The word with which it is best known comes from the name of the British scientist John Dalton (affected by “chromatic blindness”, another expression used to indicate the same disorder), who was the first to describe its fundamental characteristics in the Article “Extraordinary facts related to the vision of colors”. Color blindness affects 8% of the world population [**1**,**19**].

Blindness is a disease that is mostly congenital, linked to a genetic mutation that afflicts the X chromosome. To be able to understand the mechanisms of transmission and manifestation of color blindness it is necessary to keep in mind the concepts of genetic inheritance related to the chromosome of women and men: nature attributes two X chromosomes (XX) to women, and one X chromosome and one Y (XY) to men. 

When color blindness is hereditary, it is bilateral (affects both eyes). there are, However, there are cases in which it is monolateral. This is the case of patients who are not born with the abnormality (i.e. theirs is an extragenetic “color blindness”) but acquire it because of other diseases or conditions, such as:

- multiple sclerosis: since it is a disease affecting the cells of the nervous system, it may also involve the optic nerve or areas of the cerebral cortex that are responsible for the interpretation of visual signals;

- cataract: opacification of the lens abnormally filters light, causing partial insensitivity to blue;

- alcoholism: in alcoholics, color sensitivity is generally reduced;

- head trauma: traumatic brain damage is a condition that can reduce the ability to color discrimination;

- other eye diseases: maculopathies or other eye diseases may cause a color perception deficit.

Color blindness is caused by an alteration of the cones that influences the vision of the color self:

- red: the vision of this color may be impossible (protanopia) or difficult (protanomaly);

- green: the correct perception of green can be prevented (Deuteranopia, the type of color blindness that John Dalton suffered, and also the most common) or hindered (deuteranomaly/ teranomaly)

- blue: this color may not be perceived (tritanopia) or it is distinguished with difficulty (tritanomaly).

The diagnosis of color blindness is made by the ophthalmologist after a medical examination which consists of the recognition of colors. The best new tests are Anomaloscope of Nagel and CAD test [**2**,**21**].

The anomaloscope of Nagel is a microprocessor-controlled device for diagnosing color vision accuracy in the red/ green area (Rayleigh equation) [**3**,**4**] and the blue/ green area (Moreland equation) with integrated neutral automatic adaptation.

In its most frequent use, the principle in Rayleigh equation is based on the comparison, on a screen, of a yellow-orange reference light (lower hemisphere, wavelength of 589 nm, whose luminance can be adjusted) with a light (upper hemisphere) made up of an addition of a certain intensity of red (671 nm wavelength) and green (546 nm wavelength) [**5**-**7**]. The device is set with two knobs on the sides of the device for a normal match (yellow=15, red-green=40). 

The evaluator is asked to describe the appearance of the colors seen in the tool [**8**,**9**]. Several lots are offered for evaluation and they are asked to describe the appearance of the colors seen. The evaluator is requested to adjust the knob that controls the luminance of the test field [**10**,**11**]. This way, the centre and range of combinations are determined and recorded [**12**,**13**].

Normal subjects are sensitive to red and green and regulate these two intensities (more red than green) to obtain a yellow comparable to the reference light.

Dyschromatic “less red” subjects regulate this light only by modifying the intensity of the green, and dyschromatic “less green” by modifying the red.

## Materials and methods

The CAD test measures the size of the red/ green (RG) and/ or yellow/ blue (YB) signals needed only to see a color-defined target moving diagonally across a square made of checks that vary randomly in luminance every 50 to 80 ms. A “chromaticity chart” (CIE 1931 (x,y) - chromaticity diagram) is used for convenience and the color signal strength is measured as the distance away from the “neutral” grey background for each of the colors examined. Colored pixels have the same luminance as the gray background. During this test, the subject will see a colored target move diagonally along a central square in one of the four possible directions (top right/ left – bottom right/ left). The Response Box has 4 buttons arranged to form a square. The task of the subject is to press the right button to indicate the corresponding angle where the colored target ends and then the direction of the movement. To get a better result, the subject should be instructed to keep the view focused on the center of the square and not follow the moving target. It is designed to prevent the unjust refusal of medical certification to individuals with slight deficits in color perception [**1**-**3**].

It is important to underline that if the new limit of color, based on experiments, passed/ failed in the military requirements, 36% of the deutan subjects and 30% of the protan subjects would be classified as safe for flying.

During this test, the subject will see a colored target moving diagonally across a central square in one of four possible directions (i.e., ending top-right, top-left, bottom-right, or bottom-left). The response box provided has four buttons laid out to form a square and a central button that is not used in the CAD test. The subject’s task is to press the appropriate button (i.e., top-right, top-left, bottom-right or bottom-left) to indicate the corresponding end point and hence the direction of movement. A sharp beep follows the end of each stimulus presentation and indicates the subject to respond by pressing one of the four buttons. When unsure, the subject has to make the best guess without any hesitation. For best results, the subject should be instructed to maintain fixation on the centre of the square and not to track the moving target [**6**,**8**-**10**,**14**,**15**].

**Pilot Study**

A comparative study was conducted in dischromatopsic subjects identified during the course of the ordinary investigations directed towards the civil aero-navigating personnel by the Ophthalmology Department of the Air Force, between March 2019 and January 2020, at “Aldo Di Loreto” Institute of Aeronautical and Space Medicine of Rome. 10 subjects aged 20 to 50, with dyschromatopsia found at Ishihara’s pseudoisochromatic tables, were submitted to Oculus HMC-Anomaloscope with a manual execution program and then a CAD test. The control group consisted of 10 subjects with normal color perception, who were subjected to the same examinations. The civil aero-navigating team is subjected to its reference legislation EASA (European Aviation Safety Agency), which are the Acceptable Means of Compliance (AMC) MED B 075 of 2019 for both the first and the second class, and the Acceptable Means of Compliance ATCO MED B 075 of 2015 for the third class. While the military personnel are sent to the anomaloscope only in case of mistakes in the pseudoisochromatic tables, those sets of rules imply that the ophthalmologist must choose between one of the following tests, in order to get a second level assessment:

-anomaloscope (Nagel or equivalent);

- CAD test;

- lanterns test (spectrolux, Beynes, Holmes-Wright) [**11**,**16**].

## Discussion

In the case of civil aircrew, the reference EASA (European Aviation Safety Agency) is the acceptable means of compliance (AMC) MED B 075 of 2019 for the 1st and 2nd class and the acceptable means of compliance ATCO MED B 075 of 2015 for the 3rd class. The legislation in question for the military staff, for which only the execution of the anomaloscope in the case of errors to the pseudoisochromatic tables is previewed, obliges the examiner to choose one of the following options as a level II finding:

- anomaloscope (Nagel);

- CAD test;

- lanterns test (Spectrolux, Beynes, Holmes-Wright).

The first two examinations have been the subject of discussion during the course of this article; the tests of the lanterns, although they are still provided in the current legislation, are practically not used at national and international level because the instrumentation is technologically obsolete, outdated and no longer commercially available. The notification of Alternative Means of Compliance to the European Aviation Safety Agency by ENAC on 11.12.2019 is relevant in this regard, the National Civil Aviation Authority communicating that it will no longer accept cases whose visual requirements relating to color safety for Class 1 and Class 2 of medical certification have been determined based on the lanterns, admitting only the anomaloscope (Nagel and CAD test) [**17**,**18**,**20**].

According to a French study, both the anomaloscope with manual execution and the CAD test have high sensitivity and specificity, negative predictive value and positive predictive value identical (in both cases all values are 1.00).

The subjects in the control group were normal trichromatic in the anomaloscope test with “normal” assessment. Overlapping evidence was found in the CAD test with similar results and with “pass” assessment. The descriptive difference in the result between the two tests is inherent in the original purpose for which the two tests were designed. In fact, in the case of the anomaloscope, the instrument identifies and quantifies anomalies in chromatic perception on the red/ green axis and on the blue/ green axis, while in the case of the CAD test, the instrument not only identifies the presence of deviations in color perception but provides the operator with indications as whether or not the candidate, who had a certain color deficit, may be suitable for the civil pilot license first class, second class or achievement as an air traffic controller (CTA third class). Therefore, in this second case, the algorithm of the software is able to diversify the protocol of investigation and the sequence of the chromatic targets given depending on the class to be reached. The study led to the conclusion of the test with assessment “pass” or “fail” in the case of color perception deficit, which is not compatible with the tasks to be performed.

Therefore, it is clear that the word “pass” in the CAD test does not mean that the candidate is absolutely free of color perception deficit, but it means that even in the presence of some slight forms of dyschromatopsia, the pilot candidate or air traffic controller will be able to safely carry out all the tasks foreseen in the task to be performed. This is confirmed by the EASA regulation, which establishes the CAD test that for deuteranomaly is a threshold below the 6 standard units (SN), for protanomaly a threshold below the 12 standard units (SN) and for tritanomaly a threshold higher than the 2 standard units (SN).

On the contrary, the results of the anomaloscope should be interpreted in a more rigid way, bound to the evidence of deuteranomaly, protanomaly or tritanomaly, while the standard actually allows the operator a margin of flexibility, considering suitable the candidate with a matching range equal to or less than 4 units of scale, that is to say with a mixing light value of 40 ± 4 and a reference light of 15 ± 4.

In the light of the different construction characteristics and the different interpretation in the results of the two electro-medical products, the main question, which is the subject of this discussion, is the selective cut-off. In other words, the purpose of this article was to find out whether the two tests, considered as equivalent from the point of view of the regulation and therefore of the medical-legal ophthalmology, are also equivalent from the point of view of selection of the candidate staff. The analysis of the results in the 10 dyschromatic subjects led to the detection of 8 deuteranomalous and 2 protanomalous subjects.

As explained above, both tests showed the same sensitivity and specificity. But in practice, in selecting aero-navigating personnel, out of the two cases of deuteranomaly confirmed both to the anomaloscope, and to the CAD, the latter proved more flexible in granting the required forensic suitability. 

In fact, in the case of the anomaloscope, the matching range was far more outside the requirements of the current legislation for extremely low mixing light values in both eyes (mean values of 26.08 with an average of units of scale less than 13.9 compared to the values allowed by AMC MED EASA); in the case of the CAD test, while confirming the deuteranomaly deficiency, the threshold was found to be below the 6 normal standard units (SN) (mean value 3,9 standard units), justifying the “pass” assessment of the test.

Thus, in 2 out of 10 cases of dyschromatopsia, the anomaloscope would have guided the medical judgement, while the CAD test would have oriented towards a judgment of full fitness despite the same lack of chromatic sensitivity, which was underlined by both tests.

In conclusion, the CAD test confirmed a highly sensitive and specific method of determining the presence and depth of the chromatic perception deficit, but the method was also able to prevent the unjust refusal to certain air navigation activities of the aspirant staff. Within the framework of the study carried out, it was observed that 20% of the subjects who would have been judged unfit if subjected to the anomaloscope, have passed the CAD test and could therefore continue the certification process undertaken. Previous studies have shown that the percentages could rise to 35% in air traffic controllers (ATCO).

Moreover, the analysis of the findings suggested that in the EASA legislation currently in force at international level in the countries of the European Community showed that a rational criterion does not appear to be followed in allowing the free choice of the second level test to be administered in the dyschromatic subjects, by placing highly different tests between them on the same level, whose interpretation could discard persons who, on the contrary, if CAD tests are carried out, might have a better chance of being judged suitable [**19**,**20**].
